# Synthesis and Characterisation of Some New Aluminium Derivatives of Schiff Bases Containing N, O and S Donor Atoms and the Anti Fertility Activity of the Derivative Al[SC_6_H_4_N:C(CH_3_)CH_2_COCH_3_]_3_

**DOI:** 10.1155/S1565363303000177

**Published:** 2003

**Authors:** Simpal Sharma, Rajendra K. Sharma, Rakhi Sharma, Aruna Sharma, A. K. Rai, R. S. Gupta, Yashpal Singh

**Affiliations:** 1 Department of Chemistry University of Rajasthan Jaipur 302004 India; 2 Reproduction Physiology Section Department of Zoology University of Rajasthan Jaipur 302004 India

## Abstract

Some new compounds of aluminium having the general formula AI[SC_6_H_4_N:C(R) CH_2_ C(O)R’]_3_ where R = CH_3_, R' = CH_3_ (1); R' = CH_3_, R' = C_6_H_5_ (2); R = CF_3_, R’ = -C = CH - CH = CHS (3); R = CF_3_, R’ = C_6_H_5_ (4) have been synthesised by the reactions of Al(OPr^i^)3 and the corresponding ligands in 1:3 molar ratios in benzene. Elemental and spectroscopic (IR, ^1^H, ^13^C, and ^27^AI NMR) characterisation of these monomeric compounds reveals monofunctional bidentate behaviour of ligand moiety and the octahedral geometry around aluminium atom. Compound (1), AI[SC_6_H_4_N:C(CH_3_)CH_2_COCH_3_], has been tested for its antifertility activity in male albino rats. The oral administration of this compound at the dose level 6.5 rag/rat/day reduced the weights of testes and epididymides. Significant decrease in sperm motility as well as sperm density resulted in the reduction of male fertility by 100%. Production of primary spermatocytes (preleptotene and pachytene), secondary spermatocytes and step-19 spermatids declined by 56.10%, 44.42 %, 63.35 % and 64.57 % respectively. These results indicate that the administration of compound (1) in male rats brought about an interference with spermatogenesis which ultimately caused infertility.


### Synthesis and Characterisation of Some New Aluminium Derivatives of Schiff Bases Containing N, O and S Donor Atoms and the Anti Fertility Activity of the Derivative $Al[SC_6H_4N{:}C(CH_3)CH_2COCH_3]_3$

[a]Simpal Sharma, [a]Rajendra K. Sharma, [b]Rakhi Sharma, [b]Aruna Sharma, [a]A.K. Rai,
[b]R.S. Gupta and [a]Yashpal Singh*,

*[a]Department of Chemistry, University of Rajasthan, Jaipur-302004 (India)*
*E-mail – yp_singh07@yahoo.co.in*
*[b]Reproduction Physiology Section, Department of Zoology, University of Rajasthan, Jaipur-302004 (India)*

### ABSTRACT

Some new compounds of aluminium having the general formula $Al[SC_6H_4N{:}C(R)\ CH_2\ C(O)R']_3$ where R = $CH_3$, R′ = $CH_3$ (1); R = $CH_3$, R′ = $C_6H_5$ (2); R = $CF_3$, R′ = $\overline{-C=CH-CH=CHS}$ (3); R = $CF_3$, R′ = $C_6H_5$ (4) have been synthesised by the reactions of $Al(OPr^i)_3$ and the corresponding ligands in 1: 3 molar ratios in benzene. Elemental and spectroscopic (IR, $^1H$, $^{13}C$, and $^{27}Al$ NMR) characterisation of these monomeric compounds reveals monofunctional bidentate behaviour of ligand moiety and the octahedral geometry around aluminium atom. Compound (1), $Al[SC_6H_4N{:}C(CH_3)CH_2COCH_3]_3$ has been tested for its antifertility activity in male albino rats. The oral administration of this compound at the dose level 6.5 mg/rat/day reduced the weights of testes and epididymides. Significant decrease in sperm motility as well as sperm density resulted in the reduction of male fertility by 100%. Production of primary spermatocytes (preleptotene and pachytene), secondary spermatocytes and step-19 spermatids declined by 56.10%, 44.42 %, 63.35 % and 64.57 % respectively. These results indicate that the administration of compound (1) in male rats brought about an interference with spermatogenesis which ultimately caused infertility.

### INTRODUCTION

Organic compounds containing $-NC_6H_4S-$ unit are well known for their significant biological activities /1/. Phenothiazines significantly affect the hypothalamous pituitary gonadal axis, thus resulting in a delay in ovulation and menstruation in women /1/. In general biological activity of such type of compounds enhances considerably on complexation with metal atom /2/. Some organo-antimony(III) and –bismuth(III) compounds containing $-NC_6H_4S-$ unit have been reported to show antifertility /3/ and antitumor /4/ activity, respectively.

Benzothiazolines constitute an important class of $-NC_6H_4S-$ containing ligands. The metal derivatives of this class of ligands have been extensively pursued to investigate the chelating properties of benzothiazolines. Although mono- and bis- aluminium derivatives /5/ of benzothiazolines derived from β-diketones and 2-aminothiophenol in which the ligands behave as bifunctional tridentate /5,6/ moiety have been reported, no tris-aluminium derivatives of these ligands appear to have been synthesized so far. In view of the above, it has been considered worthwhile to synthesize a new series of tris-aluminum derivatives of benzothiazolines, and to investigate their physico-chemical and structural features as well as the antifertility activity of one of the representative compounds in male albino rats.

### EXPERIMENTAL

Moisture was carefully excluded throughout the experiments. $Al(OPr^i)_3$ was prepared by literature method /7/. Solvents (E. Merck) were dried by standard procedures /8/. Isopropanol in the azeotrope was estimated oxidimetrically /9/, using 1N $K_2Cr_2O_7$ solution in 12.5 % $H_2SO_4$. Aluminium was estimated gravimetrically as oxinate /9/. Nitrogen and sulphur were determined by Kjeldahl and Messenger methods /9/ respectively. 2-Aminothiophenol and β-diketones were distilled prior to use. The benzothiazolines /10/ RC(NH $C_6H_4S)CH_2C(O)R'$ [where R = $CH_3$, R′ = $CH_3(L^1H)$; R = $CH_3$, R′ = $C_6H_5(L^2H)$, R = $CF_3$, R′ = 2-$C_4H_3S$ ($L^3H$); R = $CF_3$, and R′ = $C_6H_5(L^4H)$] have been prepared by the equimolar condensation reactions of 2-aminothiophenol and substituted β-diketones in refluxing benzene. Liberated water was removed azeotropically with benzene. IR spectra of the ligands and their complexes have been recorded on a Nicolet DX FT IR Spectrophotometer using CsI cell. $^1H$, $^{13}C$ and $^{27}Al$ NMR spectra were recorded on a JEOL-FX-90Q (90 MHz) or Bruker DPX 300 MHz Spectrometer either in $CDCl_3$ or $C_6H_6$ solution. Molecular weights of these compounds were determined on a Knauer Vapour Pressure Osmometer in benzene solution.

Since all the complexes have been synthesised by the same method, for the sake of brevity, synthesis of one representative compound is given in detail. Synthetic and analytical results of the other compounds are summarised in Table I.

### SYNTHESIS OF COMPOUND (1)

About 25 ml benzene solution of $Al(OPr^i)_3$ (0.56 g, 2.74 mM) was added to the benzene solution (~20 ml) of the ligand $[CH_3C(NHC_6H_4S)CH_2COCH_3]$ (1.71 g, 8.25 mM) and the solution was refluxed on a fractionating column for ~4 h. The isopropanol formed in the reaction was fractionated out azeotropically with benzene. Progress as well as the completion of the reaction was checked by the estimation of the isopropanol in the azeotrope by the oxidimetric method /9/. After the completion of the reaction, the excess of the solvent was removed under reduced pressure. For the purification, this compound was dissolved in a small amount of benzene ~ 15 ml and then the petroleum ether (40-60 °C) was added slowly until a viscous compound began to separate. The solution was kept overnight at about –10 °C. The viscous compound, left after decanting off the solvent, was dried under reduced pressure. Yield, 87% (1.54 g).

**Table I**

Synthetic and analytical data of aluminium derivatives, $Al[SC_6H_4N:C(R)CH_2C(O)R']_3$.

| Reactants (g, mM) | | Molecular formula of complexes, colour, state, yield (%) and (m.p.) | $Pr^iOH$(g) found (calc.) | % Analysis Found (calc) | | | M. Wt. Found (calc) |
|---|---|---|---|---|---|---|---|
| $Al(OPr^i)_3$ | Ligand | | | Al | S | N | |
| 0.56, 2.74 | $L^1H$ 1.71, 8.25 | $C_{33}H_{36}N_3O_3S_3Al$ Reddish yellow, viscous 87 (-) | 0.47 (0.49) | 4.16 (4.18) | 14.85 (14.88) | 6.49 (6.51) | 642 (646) |
| 0.42, 2.06 | $L^2H$ 1.66, 6.16 | $C_{48}H_{42}N_3O_3S_3Al$ Pale yellow, viscous 89 (-) | 0.36 (0.37) | 3.21 (3.24) | 11.53 (11.55) | 4.99 (5.05) | 829 (832) |
| 0.31, 1.52 | $L^3H$ 1.50, 4.56 | $C_{42}H_{28}N_3O_3F_9S_6$ Pale yellow, solid 85 (92-98°C) | 0.26 (0.27) | 2.64 (2.66) | 9.46 (9.50) | 4.13 (4.15) | 1008 (1012) |
| 0.24, 1.17 | $L^4H$ 1.14, 3.53 | $C_{48}H_{33}N_3O_3F_9S_3Al$ Yellow solid 89 (95-105°C) | 0.19 (0.21) | 2.70 (2.71) | 9.65 (9.68) | 4.19 (4.23) | 990 (994) |

***Biological tests***

Male albino rats of Wistar strain weighing 150-180 g obtained from Jamia Hamdard, Hamdard University, New Delhi, were used. Animals were kept in plastic cages with proper aeration and temperature, feeding on a standard rat pellet diet (Hindustan Lever Limited) and water *ad libitum,*

After 55 days of drug administration the mating exposure test was performed in control as well as in treated groups. Rats of both groups were cohabitated with normal adult proestrous females for fertility test in the ratio of 1 : 2. Successful mating was confirmed the following morning by presence of sperms in the vaginal smear. The inseminated females were separated and number of litters delivered were recorded /11/. Proven fertile male rats were taken and divided into two groups of 10 rats each Group I → Rats received 0.5 ml olive oil for 60 days, Group II→ Rats treated with compound (1) dispersed in 0.5 ml olive oil, 6.5 mg/rat/day for 60 days.

On day 61st animals of both groups were sacrificed using ether anesthesia and their reproductive organs i.e. testes, epididymis, seminal vesicle and ventral prostate were dissected out. Half of the tissues were kept at –20 °C until assayed for biochemical estimations of protein /12/, sialic acid /13/, glycogen /14/, cholesterol /15/, and fructose /16/. The remaining half of the tissues were fixed in Bouin's fluid. Paraffin sections were made and stained with haematoxyline and eosin. The motility of cauda epididymal spermatozoa was recorded. Sperm density was assessed in testis and cauda epididymis by the method of Prasad /17/. Cell population dynamics, i.e. germinal cell counts, was based on the calculation made for each cell type per cross tubular sections. Various cell components were quantitatively analysed. Mean and standard error of mean (SEM) were calculated and the significance of difference was analysed by applying student's t-test.

### RESULTS AND DISCUSSION

Reactions of $Al(OPr^i)_3$ with benzothiazolines $[RC(NHC_6H_4S)CH_2C(O)R']$ in 1 : 3 molar ratios in refluxing benzene proceed with the rearrangement of benzothiazoline ring and result in Schiff base derivatives of aluminium.

$$Al(OPr^i)_3 + 3[RC(NHC_6H_4S)CH_2C(O)R'] \xrightarrow[\text{Reflux}]{\text{Benzene}} Al[SC_6H_4N{:}C(R)CH_2C(O)R']_3 + 3Pr^iOH$$

where $R = CH_3$, $R' = CH_3$ (1); $R = CH_3$, $R' = C_6H_5$ (2); $R = CF_3$, $R' = 2\text{-}C_4H_3S$ (3); $R = CF_3$, $R' = C_6H_5$ (4).

All the above derivatives are either coloured viscous liquids or solids and highly soluble in organic solvents like benzene, chloroform etc. These derivatives are found to be monomeric in benzene solution.

### Spectroscopic studies

### *Infrared spectra*

The absence of νSH mode (~2540 $cm^{-1}$) and the presence of νNH mode in the range 3230-3340 $cm^{-1}$ in the spectra of free ligands indicate the presence of benzothiazoline ring in the ligands. A comparative study of the spectra of complexes with corresponding ligands shows the disappearance of –NH band and the appearance of several new bands for νC=N, νAl←N [18] and νAl-S [5] in the region of 1618-1645 $cm^{-1}$, 482-502 $cm^{-1}$ and 362-439 $cm^{-1}$, respectively. This may be interpreted in terms of rearrangement of benzothiazoline ring during the formation of a Schiff base derivatives of aluminium. The spectra of the ligands as well as complexes show a band in the region 1681-1707 $cm^{-1}$ due to >C=O group. The absence of any shift in the position of this band indicates that the >C=O group does not participate in bonding during complexation.

### *$^1H$ NMR Spectra*

A comparative study of the $^1H$ NMR spectra of aluminum derivatives (Table-II) recorded using TMS as reference with the corresponding ligands shows the disappearance of the –NH signal (observed at δ 4.53 – 6.46 ppm in the spectra of ligands). This supports the rearrangement of benzothiazoline ring of the ligand during the formation of Schiff base aluminium complexes. Various R, R′ and methylene protons are observed at their expected positions with a small shift as compared to their positions in the corresponding ligands. A multiplet in the range δ 6.83 – 8.48 ppm may be assigned to the aromatic protons of Schiff bases.

### *$^{13}C$ NMR Spectra*

$^{13}C$ NMR spectra of aluminium derivatives (1-4) show C=N carbon signal in the region δ 166.82 – 167.94 ppm. This signal was observed at δ 161.39-165.74 ppm in the spectra of ligands and assigned to >C-N of benzothiozoline ring. A down field shift of ~2.0-5.0 ppm in its position reveals that this group is rearranged to >C=N on complexation and the nitrogen atom coordinates to central aluminium atom. The signals for R, R′

and $CH_2$ carbons in complexes show a small shift in comparision to their position in the free ligands. A signal for >C=O group carbon has been observed in the region δ 183.04- 190.09 ppm. No appreciable shift has been observed in the position of this signal on complexation. No shifting in the position of >C=O carbon signal indicates the non involvement of >C=O group in bonding. The aromatic carbon signals have been observed in the range δ 122.25 – 153.98 ppm (Table-III). The $C_2$ carbon of the $-NC_6H_4S-$ group shows a down field shift on complexation which further indicates the formation of an Al-S bond.

**Table II**

$^{1}H$ and $^{27}Al$ NMR Spectral data (in δ ppm) of aluminium derivatives $Al[SC_6H_4N{:}C(R)CH_2C(O)R']_3$

| Complex | R | R′ | $CH_2$ | $-NC_6H_4S-$ | $^{27}Al$* |
|---|---|---|---|---|---|
| (1) R = $CH_3$ R′ = $CH_3$ | 2.06 | 1.74 | 2.85 | 6.63-8.11 | 2.04 |
| (2) R = $CH_3$ R′ = $C_6H_5$ | 2.63 | 7.39-8.15 | 2.92 | 7.39-8.15 | 2.35 |
| (3) R = $CF_3$ R′ = 2-$C_4H_3S$ | - | 7.16-8.27 | 3.63 | 7.16-8.27 | 1.41 |
| (4) R = $CF_3$ R′ = $C_6H_5$ | - | 6.83-8.48 | 3.56 | 6.83-8.48 | 1.53 |

* Recorded in benzene solution using aluminium nitrate as standard reference.

**Table III**

$^{13}C$ NMR Spectral data (in δ ppm) of aluminium derivatives $Al[SC_6H_4N : C(R)CH_2C(O)R']_3$ in $CDCl_3$

| | Complex | R | R′ | $CH_2$ | >C=N | >C=O |  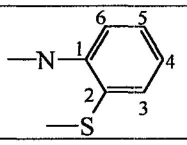 |
|---|---|---|---|---|---|---|---|
| 1. | R = $CH_3$, R′ = $CH_3$ | 29.83 | 19.86 | 57.82 | 166.83 | 190.09 | 153.17 (1), 135.48 (2), 130.32 (3), 125.76 (4), 124.93 (5), 122.25 (6) |
| 2. | R = $CH_3$, R′ =  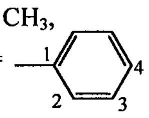 | 19.93 | 129.04 (1) 127.67 (2) 128.03 (3) 128.86 (4) | 58.01 | 166.82 | 193.59 | 153.92 (1), 136.02 (2), 132.96 (3), 126.95 (4), 125.76 (5), 122.25 (6) |
| 3. | R = $CF_3$, R′ = —C=CH—CH=CHS (4 3 2 1) | 93.44 (q) | 129.52 (1) 128.64 (2) 127.89 (3) 130.70 (4) | 65.86 | 167.35 | 183.25 | 153.11 (1), 136.40 (2), 132.72 (3), 126.68 (4), 125.46 (5), 122.44 (6) |
| 4. | R = $CF_3$, R′ =  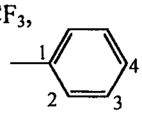 | 93.59 (q) | 130.85 (1) 128.87 (2) 128.14 (3) 129.96 (4) | 63.76 | 167.94 | 183.04 | 153.98 (1), 136.78 (2), 133.45 (3), 126.64 (4), 125.06 (5), 123.07 (6) |

### *$^{27}$Al NMR Spectra*

$^{27}$Al NMR spectra of these complexes (1-4) (Table-II) show the presence of a sharp signal in the region δ 1.41 –2.35 ppm as singlet. The appearance of $^{27}$Al NMR signal in this region confirm the existence of hexa-coordination /18/ around the central aluminium atom in all the four complexes.

In view of the monomeric nature of these complexes, the bonding of both the nitrogen and sulphur atoms of all the four ligand moieties with central aluminium atom as well as hexa-coordination as revealed by $^{27}$Al NMR, the following structure (Fig I) may be proposed for these aluminium derivatives.

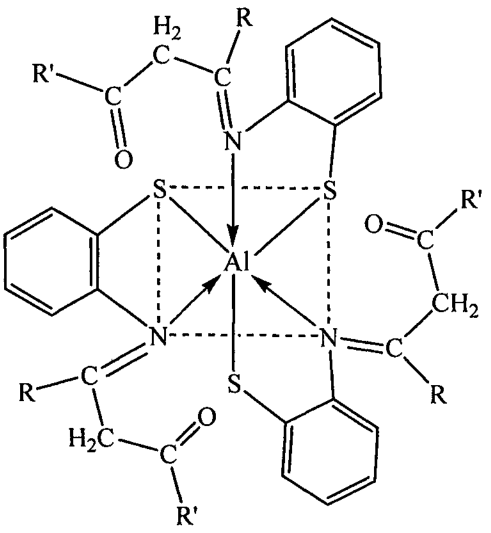

**Fig. 1:** Proposed structure for Al[SC6H4N :C(R)$CH_2$C(O)R′]$_3$ derivatives where R = $CH_3$, R′ = $CH_3$ (1); R = $CH_3$, R′ = $C_6H_5$ (2); R = $CF_3$, R′ = –C=CH-CH=CHS (3); R = $CF_3$, R′ = $C_6H_5$ (4).

It is clear from the spectral data that these ligands behave as monofunctional bidentate moiety in these derivatives. These results are in contrast to the earlier reports in which these ligands are reported to behave as bifunctional tridentate /5,6/. The monofunctional nature of the ligands in these aluminium complexes is confirmed as there is no evidence of any enolisation in these ligands and their bidentate behaviour may be easily established on the bases of maximum coordination number of aluminium, which is six, and the monofunctional nature of the ligands indicates that the enolisation of these ligands does not take place during the complexation, which is also clear from the spectroscopic evidences.

### Antifertility activity of compound Al[$SC_6H_4N$:C($CH_3$)$CH_2COCH_3$]$_3$ (1)

The body weight of rats after administration of compound (1) did not alter significantly; however the weights of testes ($P \leq 0.001$), epididymis ($P \leq 0.001$), seminal vesicle ($P \leq 0.01$) and ventral prostate ($P \leq 0.001$) were reduced significantly (Table IV). The motility of the cauda epididymal sperm as well as concentration of sperm in testes and cauda epididymis was reduced significantly ($P \leq 0.001$). The fertility of compound (1) treated rats declined up to 100 % (Table V). Most of the cell types of seminiferous tubule showed significant reduction. The population of primary spermatocytes (preleptotene and pachytene), secondary spermatocytes and step-19 spermatids declined by 56.10 %, 44.42 %, 63.35 % and 64.54 % respectively (Table VI).

**Table IV**

Effect of compound (1) on the body weights and organ weights[a]

| Treatment | Body weight (g) | Testes | Epididymides | Seminal Vesicle | Ventral Prostate |
|---|---|---|---|---|---|
| Group – I Control | 225 ± 16 | 1255 ± 26 | 525 ± 18 | 550 ± 25 | 310.5 ± 9 |
| Group – II Compound (1) treated | 180 ± 18[ns] | 1040 ± 20** | 305.5 ± 12** | 478.7 ± 22.5* | 200 ±11.5** |

[a]Values are mean ± SEM (n = 10), ns, non significant
* $P \leq 0.01$, ** $P \leq 0.001$ vs control (student's t-test)

The compound (1) treatment brings about a depletion in protein content of testes ($P \leq 0.001$), cauda epididymis ($P \leq 0.01$), seminal vesicle ($P \leq 0.01$) and ventral prostate ($P \leq 0.001$). Sialic acid content of the testes ($P \leq 0.001$), cauda epididymis ($P \leq 0.01$), seminal vesicle ($P \leq 0.01$) and ventral prostate ($P \leq 0.001$) was depleted in treated rats. Testicular glycogen was decreased to a significant ($P \leq 0.001$) degree. Level of cholesterol in testes was elevated whereas seminal vesicular fructose decreased significantly ($P \leq 0.001$) in comparison to controls (Table VII).

**Table V**

Effect of compound (1) on sperm dynamics and fertility of male rats[a]

| Treatment | Sperm Motility % (Cauda epididymides) | Sperm Density (million/ml) | | Fertility % |
|---|---|---|---|---|
| | | Testes | Cauda epididymides | |
| Control Group – I | 71.05 ± 1.85 | 4.50 ± 0.22 | 44.54 ± 2.18 | 100 % (+) ve |
| Group II Compound (1) treated | 18.45 ± 1.55** | 1.32 ± 0.25 ** | 6.50 ± 1.05** | 100 % (-)ve |

[a] Values are mean ± SEM (n = 10)
** $P \leq 0.001$ vs control (student's t-test)

The significant reduction in testes weight after the treatment with compound (1) can be attributed to the loss of germ cell /20/. It has been observed that the size of testes bears a direct relationship to the number of sperms produced /21/. The marked reduction in sperm count in our experiment also suggests a disturbed testicular and epididymal microenvironment. The treatment with compound (1) exerted a strong inhibitory effect on epididymis, seminal vesicle and ventral prostate. The decrease in weight of accessory sex organs indicates the atrophy of glandular tissue and reduction in secretory ability, thus reflecting the decreased level of testosterone /22/. It has been reported earlier that there is a high correlation of reproductive capacity with the mass of accessory sex organs /23/. Decreased seminiferous tubular diameter reflects tubular shrinkage which may occur due to cell death or sloughing of epithelial cells /24/. Sperm motility and density directly correlate with fertility and any change in them may cause infertility /25/. It is also evident that testicular function would be altered by reduced protein content /26/. Reduction of the protein content of the testes is due to loss of germ cell as well as the depletion of membrane protein content /27/.

**Table VI**

Testicular cell dynamics following compound (1) treatment[a]

| Treatment | Testicular cell counts (Number / 10 cross section) | | | | | | |
|---|---|---|---|---|---|---|---|
| | **Sertoli cell** | **Spermato-gonia** | **Prelepto-tene** | **Pachy-tene** | **Secondory spermato-cytes** | **Step – 19 Sperma-tids** | **Seminiferous tubular diameter ($\mu$m)** |
| Group – I Control | 2.98 ± 0.06 | 6.50 ± 1.9 | 20.05 ± 1.80 | 31.40 ± 1.80 | 41.10 ±2.80 | 34.02 ± 1.15 | 264.0 ± 7.0 |
| Group – II Compd. (1) treated | 2.02 ± 0.12** | 5.54 ± 0.27$^{ns}$ | 8.80 ± 1.34** | 17.45 ± 1.92** | 15.06 ± 3.02** | 12.05 ± 1.08** | 196 ± 6.5** |
| Percent deviation[b] | (-) 32.21 % | | (-) 56.10 | (-) 44.42 | (-) 63.35 | (-) 64.57 | (-) 25.75 |

[a]Values are mean ± SEM (n = 10) ns, non significant

** $P \leq 0.001$ vs control (student's t-test)

[b]Values in parentheses are percentage reduction in particular cell types.

The structural integrity of acrosomal membrane is dependent upon sialic acid and due to alteration in its content, the motility and fertilizing ability of sperms may also be effected /28, 29/. The significant decrease in glycogen content after the treatment with compound (1) possibly could be due to inhibition of glycolysis during spermatogenesis as has been observed by Bone *et al.* /30/ in ornidazole treated rats. Glycolytic metabolism of spermatozoa is also hampered by reducing level of fructose resulting in abnormal sperm function which may ultimately lead to complete male sterility /31/.

**Table VII**

Effect of Compound (1) Treatment on Biochemical Parameters of Male Rat

| | Protein (mg/g) | | | | Sialic acid (mg/g) | | | | Glycogen (mg/g) | Cholesterol (mg/g) | Fructose (mg/g) |
|---|---|---|---|---|---|---|---|---|---|---|---|
| **Treatment** | **Testes** | **Cauda epididymis** | **Seminal vesicle** | **Ventral Prostate** | **Testes** | **Cauda epididymis** | **Seminal vesicle** | **Ventral prostate** | **Testes** | **Testes** | **Seminal vesicle** |
| Group I | 205 ± 5.20 | 224.4 ± 3.27 | 215 ± 3.6 | 201.67 ± 2.22 | 4.73 ± 0.08 | 5.95 ± 0.13 | 4.95 ± 0.09 | 5.90 ± 0.10 | 3.02 ± 0.11 | 5.62 ± 0.16 | 4.56 ± 0.08 |
| Group II Compd (1) treatment | 166.5 ± 3.02** | 182.6 ± 4.05** | 189.8 ± 4.82* | 176.66 ± 4.89** | 3.69 ± 0.06** | 5.02 ± 0.15* | 4.68 ± 0.11* | 4.33 ± 0.08** | 1.66 ± 0.12** | 8.06 ± 0.25** | 2.55 ± 0.10** |

[a] Values are mean ± SEM (n = 10)

* $P \leq 0.01$, ** $P \leq 0.001$ vs control (student's t-test)

### ACKNOWLEDGEMENT

R.K. Sharma is thankful to CSIR for Senior Research Fellowship. Y. Singh and A.K. Rai are thankful to UGC for financial support.

### REFERENCES

1. R.R. Gupta, Editor, *Bioactive Molecules* (Vol. 4), Elsevier Press, Amsterdam.
2. J.R.J. Sorenson, *J. Med. Chem.*, **19**, 335 (1971).
3. R.K. Sharma, M.P. Dobhal, Y.P. Singh, A.K. Rai, R. Sharma, R. Chaudhary, R.Yadav and R. S. Gupta, *Metal Based Drugs* 7, 271 (2000).
4. P. Köpf-Maier and T. Klapötke, *Inorg. Chim. Acta*, **152**, 49(1998).
5. M. Agrawal, J.P. Tandon and R.C. Mehrotra, *Synt. React. Inorg. Met. Org. Chem*, **10**, 9 (1980).
6. M. Agrawal, J.P. Tandon and R.C. Mehrotra, *J. Inorg. Nucl. Chem.*, **43**, 1070 (1981).
7. R.C. Mehrotra, *J. Indian Chem. Soc.*, **30**, 585 (1953).
8. D.D. Perrin and W.L.F. Armarego, *Purification of Laboratory Chemicals*, 2nd ed., Pergamon Press, New York (1980).
9. A.I. Vogel, *A Textbook of Quantitative Inorganic Analysis*, Longman, London (1978).
10. WHO (19836) : Protocol MB-50 A Method for Examining the Effect of the Plant Extracts Administered Orally on the Fertility of Male Rats. APF/IP, 9914E, *World Health Organisation*, Geneva.
11. B.S. Sarswat. G. Srivastava and R.C. Mehrotra, *J. Organomet. Chem.*, **137**, 301 (1977).
12. O.H. Lowry, M.T. Rosebrough, A.L. Farr and R.J. Randall, *J. Biochem.*, **193**, 265 (1951)
13. L. Warren *J.Bio. Chem.*, **234**, 1971 (1959).
14. R. Montgomery, *Archives Biochem. Biophysics.*, **57**, 378(1957).
15. B.L. Oser, *Hawks Physiology Chemistry* (14th ed.) MC Graw Hill, New York, P. 246 (1965).
16. T. Mann, *London Metheuen and Co*, 237 (1964)
17. M.R.M. Prasad, N.J. Chinoy, K.M. Kadam, *Fertil. Steril.*, **23**, 186 (1972).
18. N. Sharma, R.K. Sharma, R. Bohra, J.E. Drakc, M.B. Hursthouse and M.E. Light, *J. Chem. Soc. Dalton Trans*, 1631 (2002).
19. S. Nagar, R.Bohra and R.C. Mehrotra, *Synt. React. Inorg. Met. Org. Chem.*, **32**, 1825 (2002).
20. U.J.A. D'Souza and K. Narayana, *Indian J. of Physiology and Pharmacology*, **45**(1), 87 (2001).
21. N. Sharma and D. Jacob, *J. of Ethnopharmacology*, **75** , 5 (2001).
22. D. Malaravizhi and P.P. Mathur, *Indian J. of Experimental Biology* (1995).
23. G. Ortiz, F. Vilchis, M. Cardenas, C. Cruz, J.C. Pedraza and M. Menjivar, *J. of Reproduction and Fertility*, **117**, 223 (1999).
24. E.D. Gol'd berg, T.G. Kaya Borovs, E.A. Timina, T.I.Fomina and V.E. Gol'd berg *Bull. Exptl. Bio/med.*, **12**, 1194 (1997).
25. M.L. Meistrich, *J. of Andrology*, **3**, 58 (1982).

26. B. Robaire and L. Hermo, *The Physiology of Reproduction.* Raven Press, New York, 999 (1988).
27. B. Barenton, M.R. Blanc, A. Caraty, M.T. Hocher-cau-de Reviers, C. Perreau and J. Soumande, *Mol. Cell. Endocrinol,* **28**, 15 (1982).
28. S.S. Riar, B.S. Setty and A.B. Kar, *Fertility and Sterility*, **24**, 353(1973).
29. H. Levinsky, R. Singer, M. Barnet, M. Sagiv, D. Allalouf. *Arch. Androl*, **10**, 45 (1983).
30. W. Bone, N.G. Jones, G. Kamp, C.H. Yeung and T.G. Cooper, *J. of Reproduction and Fertility*, **118**, 127 (2000).
31. M. Sarkar, P. Gangopdhyaay, B. Basak, K. Chakrabarty, J. Banerjji, P. Adhikary, and A. Chatterjee, *Contraception*, **62**, 271 (2000).

